# Pharmacokinetics and safety of TBAJ-587, a novel antimycobacterial diarylquinoline, in healthy participants

**DOI:** 10.1128/aac.01930-25

**Published:** 2026-07-28

**Authors:** Jerry Nedelman, Almari Conradie, Antonio Lombardi, Dean Hickman, Khalid Abd-Elaziz, Peter Dogterom, Paul Bruinenberg, Antonio J. Carcas Sansuán, Elly Sakiman, Zuzana Diamant, Jolanda van Duinen, Maria Beumont, Eugene Sun

**Affiliations:** 1 Global Alliance for TB Drug Development 486654, New York, New York, USA; 2 QPS Netherlands BV, Groningen, the Netherlands; 3 Vast Therapeutics 692781 , Morrisville, North Carolina, USA; 4 IdiPAZ, University Hospital La Paz, Servicio Madrileño de Salud (SERMAS) 16268, Madrid, Spain; 5 Department of Clinical Pharmacy and Pharmacology, University Medical Center, Groningen, the Netherlands; Queen Mary University of London, London, United Kingdom

**Keywords:** TBAJ-587, diarylquinoline, tuberculosis, pharmacokinetics, Phase 1, antimicrobial, safety

## Abstract

TBAJ-587 is a second-generation diarylquinoline with greater antimycobacterial activity and a potentially better safety profile than the first-generation bedaquiline. It is currently under development for the treatment of drug-susceptible and drug-resistant tuberculosis. A first-in-human trial of TBAJ-587, including single and multiple ascending oral doses and a dedicated food-effect cohort, was conducted in 92 healthy adults. Plasma exposures of TBAJ-587 were generally linear for AUC_tau_ and slightly subproportional for *C*_max_, with the major circulating active metabolite, M3, remaining low relative to the parent. A high-fat meal increased the mean *C*_max_ and AUC_last_ 3.46- and 2.26-fold, respectively. TBAJ-587 accumulated with multiple dosing over the 28-day period, with mean accumulation ratios across the three tested doses ranging from 1.69 to 2.32 for *C*_max_ and from 2.74 to 3.73 for AUC_tau_. However, steady-state conditions were not yet reached on day 28. Mean terminal half-lives after 28-day dosing of TBAJ-587 ranged from approximately 80 to 111 days. There were no deaths or serious adverse events, and TBAJ-587 was generally safe and well tolerated at single doses of 25–800 mg under fasting conditions and multiple doses of 50–200 mg once daily for 28 days after a standard breakfast. In addition, no dose- or time-dependent effects were noted for any of the other safety and tolerability parameters, including no clinically significant effects on the QTc interval. These results support further investigation of TBAJ-587 for the treatment of tuberculosis.

**CLINICAL TRIALS:**

This study is registered with ClinicalTrials.gov as NCT04890535.

## INTRODUCTION

In 2024, around 10.7 million people globally fell ill with tuberculosis (TB), with an estimated 1.23 million deaths (1). Current treatment regimens for drug-susceptible TB (DS-TB) are efficacious but lengthy, often leading to non-adherence, resulting in poor clinical outcomes and development of drug resistance (2). Drug-resistant TB (DR-TB), particularly resistance to rifampicin, a first-line drug for treatment of DS-TB, continues to be a public health threat (1).

Bedaquiline (BDQ) is a first-in-class diarylquinoline (DARQ) drug approved in 70 countries for combination therapy in adults with pulmonary multidrug-resistant (MDR) TB. Although BDQ has become an important component of MDR-TB treatment (3), it has safety issues, including prolonged QT interval, cardiotoxicity, and hepatotoxicity (4). Furthermore, resistance to BDQ is emerging (5, 6).

TBAJ-587 is a novel DARQ antibiotic under development for the treatment of both DS- and DR-TB. Like BDQ, TBAJ-587 selectively inhibits ATP synthase, an enzyme essential for the generation of energy in TB mycobacteria, but with 4- to 7-fold greater potency in wild-type mycobacteria (7) and similar superiority in resistant strains (data on file). Similarly, in a murine TB model, TBAJ-587 demonstrated greater bactericidal activity in both wild-type and resistant strains and improved the efficacy of combination regimens while limiting emergence of resistance (8). Substitution of BDQ with TBAJ-587 in combination regimens in mice significantly enhanced sterilizing activity, shortening the treatment duration required for cure (9).

In pre-clinical models, TBAJ-587 has three circulating metabolites, M2, M3, and M12. M3 is the N-desmethyl metabolite analogous to BDQ’s M2. M3 is approximately as potent against wild-type TB mycobacteria as BDQ. M2 and M12 are O-desmethyl and di-desmethyl metabolites, respectively. Plasma concentration ratios of metabolites to parent vary across species, with M3 approaching equivalence to parent in mice and dogs, and M2 and M12 ratios ranging from 0.01 to 0.4.

Pre-clinical safety evaluations have demonstrated acceptable toxicologic and tolerability profiles *in vitro* and in rats and dogs, including a reduced risk of QT prolongation compared with BDQ and its corresponding metabolites. The most concerning potential adverse effect, a determining factor for dose selection in the present trial, was cardiac damage to either myocardium or valve leaflets, which was seen with repeated daily administration in dogs at doses higher than the NOAEL of 10 mg/kg/day, at which dose level the ultimate values of AUC_0–24h_ were 53,600 and 60,000 h·ng/mL for TBAJ-587 and M3, respectively.

Here, we present the results of a two-part, phase 1 study of TBAJ-587 in healthy adults. In Part 1, the safety and pharmacokinetics (PK) (including high-fat-meal effect) of single-ascending doses (SAD) of TBAJ-587 were investigated. In Part 2, multiple-ascending doses (MAD) given once daily after the intake of a standard breakfast for 28 days were investigated.

## RESULTS

### Participants

Ninety-two participants were enrolled (see Fig. S1 for disposition). Some cohorts were not fully enrolled due to recruitment challenges posed by the COVID-19 pandemic. All SAD doses were administered under fasting conditions. All MAD doses were administered after consumption of a standard breakfast, based on the pharmacokinetic outcomes of the food-effect (FE) cohort.

All SAD and FE participants completed the trial as per protocol. All MAD participants completed dosing, and three participants in the 50 mg MAD cohort withdrew consent for personal reasons after receiving all planned doses and continuing 28–112 additional days through follow-up. Withdrawing consent was not related to any adverse events.

Demographic characteristics of the participants by trial and dose group are shown in Table 1.

**TABLE 1:** Participant characteristics

Arm	*N*	Age (years)	Weight (kg)	Female	Not Hispanic or Latino^*a*^	White^*b*^
		Mean (range)	Mean (range)	*N* (%)	*N* (%)	*N* (%)
SAD						
Placebo	12	45.1 (22–60)	79.9 (64.2–97.2)	0	12 (100)	9 (75)
25 mg	6	28.3 (22–36)	79.8 (65.9–104)	0	6 (100)	6 (100)
50 mg	4	31.0 (20–50)	86.4 (65.8–113)	0	4 (100)	4 (100)
100 mg	5	28.2 (22–36)	77.3 (69.6–83.5)	0	5 (100)	5 (100)
200 mg	6	44.5 (32–57)	79.9 (69.8–95.4)	1 (16.7)	6 (100)	6 (100)
400 mg	6	45.5 (31–55)	82.2 (72.4–90.5)	1 (16.7)	5 (100)	3 (50)
800 mg	6	46.0 (19–59)	82.9 (71.8–95.2)	1 (16.7)	5 (83.3)	5 (83)
Food effect						
200 mg fed	9	51.1 (33–63)	79.4 (53.1–95.2)	2 (22.2)	8 (88.9)	9 (100)
200 mg fasting	8	43.9 (31–57)	77.7 (67.9–95.4)	1 (12.5)	8 (100)	8 (100)
MAD						
Placebo	9	44.9 (23–63)	75.5 (62.5–103)	2 (22.2)	8 (88.9)	9 (100)
50 mg	9	46.3 (28–63)	81.7 (60.2–92.9)	1 (11.1)	9 (100)	7 (77.8)
100 mg	9	41.4 (23–63)	77.2 (71.6–83.7)	1 (11.1)	9 (100)	9 (100)
200 mg	9	50.8 (28–64)	75.5 (59.9–84.9)	2 (22.2)	9 (100)	9 (100)

^*a*^ Not Hispanic or Latino is a category of ethnicity; the complement is Hispanic or Latino.

^*b*^ White is a category of race. Other categories represented were American Indian or Alaska Native (SAD: 1), Asian (SAD: 4, MAD: 1), Black or African American (MAD: 1), and other (SAD: 2).

### Pharmacokinetics

**Fig 1:**
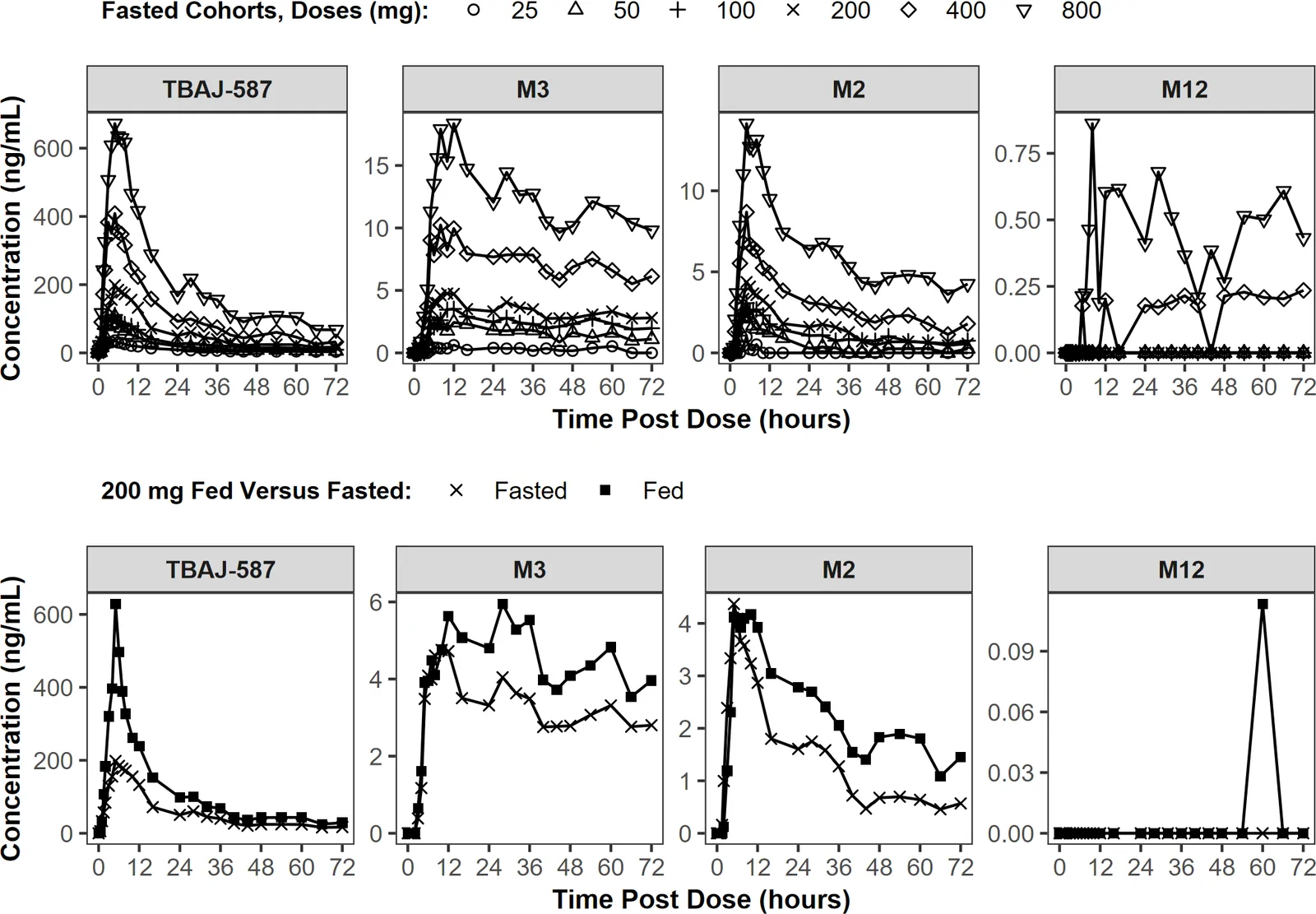
Mean plasma concentrations of TBAJ-587 and its metabolites following a single dose of 25, 50, 100, 200, 400, and 800 mg TBAJ-587 oral suspension in healthy adults under fasted conditions and comparing fasted and fed conditions for the 200 mg dose.

As seen in Fig. 1, after single doses, TBAJ-587 and its metabolites exhibited multiphasic pharmacokinetic profiles with long terminal half-lives. Exposure increased with dose; see Table 2 for TBAJ-587 and Table S2 for M3. Although Fig. 1 shows concentrations only through 72 h post-dose for convenient visualization, PK in the SAD part was monitored through 126 days after dosing. Concentrations of TBAJ-587 were quantifiable (>1 ng/mL) through 126 days in some participants at every dose except 25 mg and in all participants at doses of 200 and 800 mg. However, in only a minority of participants were the slopes of the profile tails stable enough for estimation of half-life and AUC_inf_. Therefore, these parameters’ behavior could not be determined meaningfully.

**TABLE 2:** SAD and FE pharmacokinetic parameters for TBAJ-587^*a*^

Arm	*N*	*T*_max_ (h)	*C*_max_ (ng/mL)	AUC_last_ (h*ng/mL)
		Median (range)	Mean (SD)	Mean (SD)
SAD (oral suspension and fasting)				
25 mg	6	5 (4–5)	36.7 (5.82)	1,880 (468)
50 mg	4	5 (5–5)	106 (33.8)	7,250 (4,390)
100 mg	5	5 (3–8)	114 (41.7)	12,400 (7,260)
200 mg	6	5 (2–8)	224 (126)	20,100 (12,200)
400 mg	6	5 (5–5)	409 (205)	36,100 (22,400)
800 mg	6	5 (4–7)	699 (258)	61,200 (20,200)
Food effect (oral suspension)				
200 mg fed	9	5 (5–6)	632 (114)	37,000 (6,330)
200 mg fasted	8	5 (2–8)	208 (116)	18,600 (11,000)

^*a*^ AUC_last_, area under the concentration-time curve from zero to the last quantifiable sample collection time; *C*_max_, maximum (peak) plasma concentration; SD, standard deviation; *T*_max_, time to maximum (peak) plasma concentration.

Inferential analyses of dose proportionality in the SAD part for *C*_max_ and AUC_last_ are summarized in Tables S5 and S6 for TBAJ-587 and M3, respectively. *C*_max_ increased less than dose proportionally, while AUC_last_ increased approximately dose proportionally for TBAJ-587 and more than proportionally for M3 .

Dosing with a high-fat meal increased exposure for TBAJ-587 (Table S7) and M3 (Table S8). However, food reduced relative variability in that although mean values of *C*_max_ and AUC_last_ increased with food for both analytes, standard deviations either decreased or remained about the same (Table 2 and Table S2). Whereas in the FE cohort an FDA standard high-fat, high-calorie breakfast was consumed, in the MAD part of the trial, daily doses were administered approximately 30 min after a standard breakfast. The two menus are listed in Table S14. Relative to the high-fat meal, the standard breakfast produced slightly lower parent exposure after a single 200 mg dose (Table S15a and b), but it still increased exposure relative to fasting (Table S15c).

**Fig 2:**
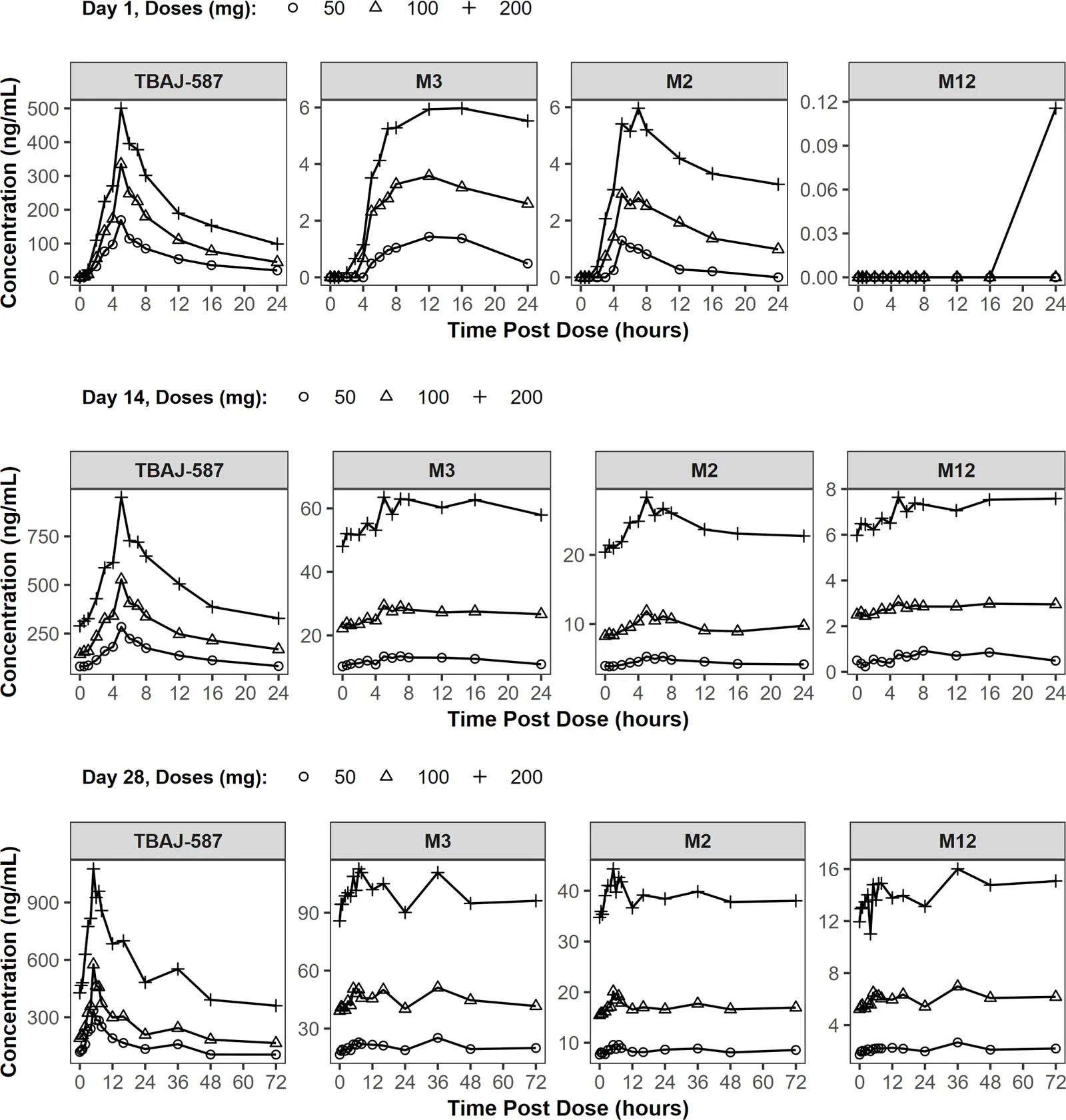
Mean plasma concentrations of TBAJ-587 and its metabolites following multiple doses of 50, 100, and 200 mg TBAJ-587 oral suspension in healthy adults under fed conditions.

In the MAD part, mean *C*_max_ and AUC_tau_ of TBAJ-587 and M3 increased with dose and days of dosing (Fig. 2; Table 3 and Table S4).

**TABLE 3:** MAD pharmacokinetic parameters for TBAJ-587^*a*^^*,^b^*^

Arm	*N*	*T*_max_ (h)	*C*_max_ (ng/mL)	AUC_tau_ (h*ng/mL)	*R*_Cmax_	*R*_AUC_	*T*_1/2_ (h)
		Median (range)	Mean (SD)	Mean (SD)	Mean (SD)	Mean (SD)	Mean (SD)
Day 1							
50 mg	9	5 (5–5)	169 (74.9)	1,340 (483)			
100 mg	9	5 (5–6)	352 (103)	2,710 (661)			
200 mg	9	5 (5–7)	513 (176)	4,720 (802)			
Day 14							
50 mg	9	5 (5–6)	288 (109)	3,280 (904)	1.82 (0.533)	2.63 (0.908)	
100 mg	9	5 (5–7)	528 (226)	6,250 (2,280)	1.56 (0.648)	2.25 (0.411)	
200 mg	9	5 (5–5)	950 (341)	11,800 (3,690)	1.95 (0.727)	2.49 (0.594)	
Day 28							
50 mg	9	5 (5–6)	338 (96.9)	4,610 (1,230)	2.22 (0.784)	3.73 (1.37)	2,340^c^ (699)
100 mg	9	5 (5–5)	576 (179)	7,520 (2,550)	1.69 (0.429)	2.74 (0.458)	2,670^d^ (985)
200 mg	9	5 (5–16)	1,110 (461)	16,900 (7,350)	2.32 (1.06)	3.57 (1.35)	1,930^e^ (907)

^*a*^ *T*_1/2_ reported only for day 28, where sampling continued beyond 24 h post-dose; adjusted *R*^2^ value <0.80: *^c^N *= 5; *^d^N *= 7; *^e^N *= 6.

^*b*^ AUC_tau_, area under the curve limited to the end of a dosing interval; *C*_max_, maximum (peak) plasma concentration; *R*_AUC_, AUC accumulation ratio as compared to the first fed day; *R*_Cmax_, *C*_max_ accumulation ratio as compared to the first fed day; SD, standard deviation; *T*_1/2_, terminal-phase elimination half-life; *T*_max_, time to maximum (peak) plasma concentration.

Dose proportionality for TBAJ-587 could not be rejected for both *C*_max_ and AUC_tau_, as the 90% CIs for *β* included one for all assessments (Table S11). However, point estimates for *β*, especially for *C*_max_ (0.833–0.864), suggest the possibility of somewhat less than proportional increases in exposure with increasing doses. Similarly, M3 exhibited approximate dose proportionality, although on days 14 and 28, lower limits of the confidence intervals for *β* were close to 1, suggesting the possibility of somewhat greater than proportional increases in exposure (Table S12).

Exposure of TBAJ-587 and M3 increased up to 4-fold (Table 3) and 20-fold (Table S4), respectively. Mean metabolite-to-parent ratios increased over time (Table S13).

After 28 days of multiple dosing, mean values of *T*_1/2_ that could be calculated for TBAJ-587 ranged from 1,930 to 2,670 h (approximately 80–111 days) (Table 4). Results were similar for M3 (Table S4). Consistent with these values, pre-dose concentrations exhibited continued, slow accumulation through day 28, and steady state was not yet reached by day 28 (Fig. S2 and S3).

**TABLE 4:** Mean QTcF intervals and change from baseline

Group	*N*	QTcF (ms)	ΔQTcF (ms)
		Mean (SD)	Mean
SAD and FE, day 1, 5 h post-dose			
Placebo	12	407 (17.2)	−4.20 (14.7)
25 mg	6	410 (7.87)	−10.3 (5.88)
50 mg	4	412 (21.9)	−6.08 (16.1)
100 mg	5	402 (17.4)	0.18 (7.46)
200 mg fasted	8	402 (11.4)	−6.26 (12.4)
200 mg fed	9	402 (16.1)	−8.97 (11.5)
400 mg	6	405 (16.9)	−17.6 (18.3)
800 mg	6	410 (6.40)	−7.98 (12.3)
MAD, Day 28, 6 h post-dose			
Placebo	9	408 (21.1)	−8.32 (7.80)
50 mg	9	409 (16.2)	−10.4 (11.0)
100 mg	9	401 (20.6)	−15.6 (4.37)
200 mg	9	403 (19.4)	−9.56 (10.9)

### Tolerability and safety

TBAJ-587 was well tolerated with an acceptable safety profile in all parts of the trial. There were no deaths or serious adverse events or treatment-emergent adverse events (TEAEs) that resulted in trial discontinuation. Most TEAEs were mild or moderate in intensity, and almost all events resolved. (The following events were still unresolved at the end of follow-up: hypophosphatemia (N=4), elevated CPK (N=2), and 1 case each of decreased ANC count, flu-like symptoms, sleepiness, tinnitus, and dry scalp. None of them were deemed related to study drug.) The overall prevalence of TEAEs was similar in the active and placebo groups and did not show a dose or time dependence. The most common TEAE in both parts of the study was headache, most often deemed possibly related to the study drug. The most common laboratory abnormalities were elevated creatine phosphokinase (CPK) and hypophosphatemia, which are described in greater detail below and in the Supplemental material. Table S16 summarizes grade 2 and higher TEAEs. Table S17 summarizes TEAEs of all grades that occurred in at least two participants.

There were three grade 4 TEAEs among participants on TBAJ-587 and none among participants on placebo. All three events were asymptomatic and assessed as unrelated to the study drug by the investigator. More details are provided in the Supplemental material.

Grade 3 TEAEs occurred in both placebo and active groups. More details are provided in the Supplemental material.

CPK was of interest because of pre-clinical findings of muscle toxicity in rats. Some participants (5 in the SAD and 4 in the MAD) were reported with asymptomatic CPK elevations. All but one of the events occurred after dosing, were associated with some type of intense physical activity, and were deemed unrelated to TBAJ-587. The one exception was an increased CPK grade 3 event that occurred while on treatment on study day 14 in a participant receiving 100 mg in the MAD. The elevation resolved promptly without intervention. In 2 participants with CPK elevations, CPK-MB and AST were also increased while troponin T was normal. These laboratory abnormalities were completely asymptomatic and not associated with ECG changes, suggesting a non-cardiac origin for these enzyme elevations. More details on CPK elevations are provided in the Supplemental material.

No clinically significant abnormalities in cardiac troponin T as a marker for cardiotoxicity were observed.

Hypophosphatemia was of interest because it was also observed pre-clinically in rats. In the SAD part, four participants were reported with TEAEs of hypophosphatemia, mostly grade 3, except for one participant with two events of grade 4 decrease on study days 56 and 126, respectively. In the MAD part, one participant experienced grade 3 hypophosphatemia on Study Day 168. All participants with hypophosphatemia TEAEs were asymptomatic. More details are provided in the Supplemental material.

No clinically significant alterations or dose- and/or time-dependent effects were observed in vital signs and ECG parameters.

Table 4 summarizes QTcF values and changes from baseline around *T*_max_ for TBAJ-587. There were no meaningful differences between any of the single or multiple dosing regimens of TBAJ-587 and placebo.

## DISCUSSION

In this first-in-human trial, the DARQ TBAJ-587 was well tolerated and had an overall acceptable safety profile and demonstrated predictable PK in healthy adult participants, supporting its continued development as a next-generation ATP synthase inhibitor for tuberculosis. TBAJ-587 was designed to address several limitations of BDQ, including QT prolongation and the emergence of resistance driven by mutations in *atpE* and *Rv0678*.

TBAJ-587 displayed a multiphasic PK profile with slow absorption, with median *T*_max_ consistently near 5 h following both single and multiple doses. The long terminal half-life observed after 28 days of daily dosing (approximately 80–111 days) appears slightly shorter than BDQ’s half-life of approximately 5.5 months (4) but nonetheless suggests that steady state will not be achieved with treatment durations of 6 months, standard now for TB therapy, or shorter, aspirational for new regimens with TBAJ-587. Across 28 days of once-daily dosing, exposure of TBAJ-587 accumulated 2- to 4-fold, while its major metabolite M3 accumulated 15- to 20-fold, reflecting the slower appearance of M3 on Day 1 (small denominator). Mean metabolite-to-parent ratios increased over time, consistent with accumulation and progressive formation of metabolites during repeated dosing (Table S13). Selection of doses for longer studies in patients will benefit from PK modeling to predict additional accumulation (10).

Dose-exposure relationships for TBAJ-587 were generally linear for AUC and slightly subproportional for *C*_max_. M3 demonstrated greater than proportional increases in the SAD part, which may reflect non-linear formation or elimination. Nevertheless, metabolite exposures remained low relative to parent drug and substantially lower than in pre-clinical species used to study toxicology.

A pronounced food effect was observed with a high-fat meal, producing higher and relatively less variable exposures for both TBAJ-587 and M3. These results are consistent with enhanced absorption of a lipophilic DARQ under fed conditions and supported the selection of a practical fed dosing condition for multiple dosing. A standard breakfast, used in the MAD part, resulted in exposures between fasting and high-fat conditions and is likely sufficient for routine clinical dosing, avoiding the logistical burden of a high-fat meal at every dose, though standardized meal timing may be needed to minimize PK variability.

Across SAD and MAD cohorts, TBAJ-587 was generally safe and well tolerated. No treatment-related serious adverse events, study-drug-related withdrawals, or clinically significant trends in vital signs or ECG parameters were observed. Notably, no QTcF prolongation signals were detected, addressing a key limitation of BDQ. Laboratory abnormalities were limited primarily to asymptomatic elevations in CPK and asymptomatic, transient hypophosphatemia, which were consistent with pre-clinical findings. Grade 4 events, two CPK elevations and one hypophosphatemia, were not time- or dose-dependent and were assessed as unrelated to the study drug. The delayed timing of several CPK events, occurring weeks after the last dosing, suggests a non-drug-related etiology but does not prove it because of the drug’s long half-life. Of note, all but one of the CPK elevations reported in the study occurred during the follow-up period and were associated with prior intense physical activity. The absence of myocardium- or valve-associated adverse events is consistent with the exposure margins chosen based on the canine NOAEL. While all CPK and phosphate abnormalities were asymptomatic and not associated with other clinical adverse events, they will continue to be investigated in future clinical studies of greater size and longer duration.

One limitation of this phase 1 trial is that most participants were white males. Women of childbearing potential were excluded. Future studies are planned in a more diverse cohort of TB patients.

Overall, this trial supports continued investigation of TBAJ-587 as a second-generation DARQ for TB treatment, with careful dose selection for longer-term studies that accounts for prolonged accumulation, close monitoring of selected AEs, and further research on long-term PK in various subpopulations.

## MATERIALS AND METHODS

### Trial design

TBAJ-587-CL-001 was a phase 1, partially blinded, randomized, placebo-controlled trial with two parts. The sponsor trial team, safety review committee, all participants, and clinical staff were blinded. The pharmacy staff was unblinded, as were two independent reviewers whose feedback was included in dose-escalation decisions. Part 1 had a SAD design in six fasting cohorts and an additional cohort for the assessment of the effect of a high-fat meal on the PK of TBAJ-587 (FE Cohort). Part 2 had a MAD design with three cohorts dosed once daily after the intake of a standard breakfast for 28 days.

### Exclusion criteria

Participants were excluded if they were younger than 18 years or were 65 years of age or older; had a body mass index outside the range of 18.5–32 kg/m^2^ or weight <50 kg; were females of childbearing potential; had a history of clinically relevant cardiovascular, musculoskeletal, pulmonary, hepatic, renal, hematological, gastrointestinal, endocrine, immunological, dermatological, neurological, or psychiatric disease or heart murmur, any clinically significant laboratory abnormalities, including elevated ALT, AST, total bilirubin, creatinine, creatine phosphokinase, pancreatic amylase or lipase, or positive hepatitis B, hepatitis C, or HIV; had severe CoV-2 infection at screening; alcohol and/or substance abuse within the past 2 years; recent tobacco use (positive urine cotinine); had an exposure to prescription medications within 14 days prior to the first dose, over-the-counter medications, herbal medications, or vitamin supplements except acetaminophen within 7 days prior to the first dose; had a finding of clinically significant ECG abnormalities or a finding of abnormal pulse or blood pressure on repeated testing at screening; had a QTcF interval (QT interval corrected for heart rate by Fridericia’s formula) of >450 ms for males or >470 ms for females at screening, day −1, or day 1 (pre-dose), or a history of prolonged QT syndrome; or had family history of prolonged QT syndrome or unexplained sudden death. Participants were also excluded if they had used any significant inhibitors of CYP enzymes and/or significant inhibitors or substrates of P-gp and/or organic anion transporting polypeptides within 14 days prior to the first dose of TBAJ-587, or any inducers of CYP enzymes and/or P-gp, including St. John’s Wort, within 30 days prior to the first dose.

### Part 1: SAD and FE

In each of the six SAD cohorts, participants were randomized between placebo (planned *n* = 2 per cohort) and one of the doses, 25, 50, 100, 200, 400, or 800 mg of TBAJ-587 (planned *n* = 6 per cohort). TBAJ-587 and placebo were administered as an oral suspension formulation under blinded conditions after a fast of at least 10 h. Oral suspensions of TBAJ-587 were formulated at concentrations of 5 and 20 mg/mL to accommodate dosing requirements across trial cohorts. For the SAD cohort receiving 800 mg, the maximum administered volume was 40 mL, corresponding to the highest dose evaluated in the trial. After dosing, fasting continued for at least 4 h, whereafter regular meals were taken according to the procedures of the clinical site. Only the first cohort followed a sentinel approach: initial dosing of one participant on TBAJ-587 and one participant on placebo. After an evaluation period of at least 72 h, the remaining participants of that cohort were dosed.

Dose escalation decisions were based on a blinded review of safety and tolerability data collected over a period of at least 14 days and PK data collected over at least 7 days.

Based on observed PK and safety/tolerability results, the 200 mg dose was selected to assess the effect of food on the PK of TBAJ-587 in a parallel-arm design. For the fasting arm, it was planned to add three additional participants to the six participants who had already received 200 mg of TBAJ-587 in the corresponding SAD cohort. The fed arm was planned to contain nine additional participants to receive 200 mg TBAJ-587 after the intake of a high-fat meal.

The participants’ confinement period lasted from day −2 up to day 8, after which participants were followed up with frequent ambulatory visits for PK sampling until day 126.

### Part 2: MAD

Within each of the three MAD cohorts, participants were randomized between placebo (*n* = 3 per cohort) and one of the doses, 50, 100, or 200 mg of TBAJ-587 (*n* = 9 per cohort). TBAJ-587 and placebo were administered after consumption of a regular breakfast as an oral suspension formulation under blinded conditions, once daily for 28 days. The participants’ confinement period lasted from day −2 up to day 31, after which participants were followed up with frequent ambulatory visits for PK sampling until day 168.

Dose escalation decisions were based on a blinded review of safety and tolerability data collected over a period of at least 21 days and PK data collected over at least 14 days.

### Dose selection

#### Part 1: SAD and FE

Considerations of both efficacy and safety influenced the selection of doses in part 1. Doses were sought for TBAJ-587 that would yield at least comparable and preferably superior efficacy to BDQ. A PK model was developed using non-clinical PK data and scaled to humans. Using the model, efficacy was predicted for different TBAJ-587 doses by matching AUC/MIC for TBAJ-587 and M3 to the corresponding ratio for BDQ and under the assumption of daily dosing for TBAJ-587. Comparisons between efficacy achieved in mouse models of TB, following dosing of BDQ or TBAJ-587, were also considered. The doses of TBAJ-587 that were projected to yield comparable efficacy to BDQ ranged from 25 to 50 mg daily, with doses higher than 50 mg projected to result in superior efficacy.

The starting dose of 25 mg was judged acceptable because, based on body surface area extrapolation, it was at least 1/13th of the human equivalent dose of the no-observed-adverse-effect level (NOAEL) for the most sensitive non-clinical species (dogs). The maximum dose level of 800 mg was judged acceptable because projected exposures of TBAJ-587 were at least sixfold lower in humans than at the NOAELs and the lowest adverse effect levels (LOAELs) in animals.

#### Part 2: MAD

Doses for part 2 were selected based on comparisons with exposures in the 3-month dog repeat-dose toxicology study. Because of the 15-fold higher plasma exposures of M3 in the dog toxicology study relative to humans, the metric of exposure for comparison was the sum of the AUC_0–24_’s of TBAJ-587 and M3 (SAUC). The mean SAUC at the end of 3 months in the dog study at the NOAEL dose, 10 mg/kg, was 113.6 h*μg/mL. A PK model fitted to the data from part 1 predicted that a dose of 200 mg administered daily under fed conditions would yield 18.5 h*μg/mL as the SAUC after 28 days. This predicted exposure is lower than the mean exposure at the dog study’s NOAEL, while 200 mg is four to eight times higher than the 25–50 mg range of doses predicted to be as efficacious as BDQ, as discussed above. Two hundred milligrams was selected as the high dose for the MAD, and 50 mg as the low dose.

### Assessments

#### Safety and tolerability monitoring

Safety assessments were performed at time points defined in the trial protocol. For all cohorts, physical examination (including heart murmurs), vital signs, ECGs, extensive cardiac monitoring, 24-h Holter monitoring (except for the fed arm of the FE cohort, performed to continuously monitor cardiac activity for intermittent abnormalities), adverse events (AEs), hematology, serum chemistry, coagulation, and urinalysis were used to assess safety and tolerability. Blood and urine were collected for additional clinical laboratory evaluations including cardiac troponin T, CPK, and CPK-MB because of pre-clinical findings of myocardial and skeletal muscle toxicity previously discussed. Female participants had pregnancy testing during screening and at check-in.

#### Blood sampling for PK

In part 1, sampling for PK occurred at pre-dose and at 0.5, 1, 1.5, 2, 3, 4, 5, 6, 7, 8, 10, 12, 16, 24, 28, 32, 36, 40, 44, 48, 54, 60, 66, 72, 78, 84, 90, 96, 108, 120, 132, 144, 156, and 168 (=day 8) h post-dose. Additional sampling was scheduled for days 9, 10, 11, 12, 13, 14, 21, 28, 42, 56, 70, 84, 98, 112, 126, and early withdrawal. However, sampling could be terminated early if observed concentrations dropped below the quantification limit. In the MAD part, pre-dose trough concentrations were collected daily through day 28. On days 1, 14, and 28, a post-dose profile was collected at 0.5, 1, 2, 3, 4, 5, 6, 7, 8, 12, 16, and 24 h. Additional post-treatment samplings were obtained on days 30, 31, 35, 42, 56, 70, 84, 98, 112, 126, 140, 154, 168, and early withdrawal, if applicable.

### Bioanalytical procedures

The determination of TBAJ-587, M2, M3, and M12 in human EDTA plasma was done using LC-MS/MS by the bioanalytical facility of QPS Netherlands.

The assay comprised protein precipitation, followed by analysis of the extracts using LC-MS/MS. Before processing, the samples were spiked with an internal standard. The analyte and respective internal standard were validated in MRM mode. Analyte-to-IS peak area ratios for the standards were used to obtain a linear calibration curve using 1/*x*^2^ weighted least squares regression analysis.

The linearity, sensitivity, selectivity, spike-in selectivity, effect of lipemic plasma, effect of hemolytic plasma, precision, accuracy, matrix effect, recovery, dilution, batch size, and carryover were evaluated. The LC-MS/MS bioanalytical method for determination of the concentration of TBAJ-587 and its metabolites (M2, M3, and M12) in human EDTA plasma was validated by QPS. For all analytes, the ULOQ of the assay was 1,000 ng/mL, and the LLOQ was 1.00 ng/mL.

### Pharmacokinetic and statistical evaluation

Standard non-compartmental PK parameters were calculated for TBAJ-876 and M3 using Phoenix WinNonlin (version 8.3.4.295, Certara, L.P.). *C*_max_ and *T*_max_ were directly determined from the observed plasma concentration data. The observed terminal elimination rate constant (*λ*_*z*_) was calculated by linear regression of the log concentration in the apparent terminal elimination phase of the concentration time profile. Points prior to *C*_max_ (including *C*_max_) were not used. During the analysis, regressions using the last three points with non-zero concentrations, then the last four points, and the last five points, and so forth, were repeated. For each regression, an adjusted coefficient of determination (adjusted *R*^2^) was computed. *λ*_*z*_ was estimated using the regression with the highest adjusted *R*^2^ ≥0.8 and, if the adjusted *R*^2^ did not improve but was within 0.0001 of the largest adjusted *R*^2^ value, the regression with the larger number of points was used.

Statistical assessments were performed on TBAJ-587 and M3 using SAS (version 9.4; SAS Institute, Inc.).

Dose proportionality for exposure parameters (*C*_max_ and AUC) was assessed using a power model approach (11). All tests were performed using a two-sided α-level of 0.05, and appropriate confidence intervals were provided.

The effect of food was assessed by statistical comparison of the PK parameters of TBAJ-587 and M3 using an analysis of variance model for a parallel group design on the ln-transformed data with treatment as a fixed effect. Conclusions regarding the results of the statistical analysis of PK parameters across treatments were based on the ratio of the geometric means expressed as a percentage and the 90% CI about the ratio.

Safety parameters were presented descriptively, and change from baseline was evaluated for laboratory safety, vital signs, and ECGs.

## Data Availability

Study data sets are available from the corresponding author upon reasonable request.

## SUPPLEMENTAL MATERIAL

Supplemental material aac.01930-25-s0001.docx Fig. S1 to S7; Tables S1 to S17.

ASM does not own the copyrights to Supplemental Material that may be linked to, or accessed through, an article. The authors have granted ASM a non-exclusive, world-wide license to publish the Supplemental Material files. Please contact the corresponding author directly for reuse.
