# Supplementary material for: Pharmacokinetics and safety of TBAJ-587, a novel antimycobacterial diarylquinoline, in healthy participants
Source: Antimicrob Agents Chemother. 2026 Jul 28;70(9):e01930-25. doi: 10.1128/aac.01930-25 (PMC13712999; doi:10.1128/aac.01930-25)
Supplement: Supplementary file 1 [file aac.01930-25-s0001.docx]

Supplementary Material for: Pharmacokinetics and safety of TBAJ-587, a novel antimycobacterial diarylquinoline, in healthy participants

Jerry Nedelman^a,#^, Almari Conradie^a^, Antonio Lombardi^a^, Dean Hickman^a^, Khalid Abd-Elaziz^b^, Peter Dogterom^b^, Paul Bruinenberg^c^, Antonio J. Carcas Sansuán^d^, Elly Sakiman^b^*,* Zuzana Diamant^e^, Jolanda van Duinen^b^, Maria Beumont^a^, Eugene Sun^a^

^a^Global Alliance for TB Drug Development, New York, New York, USA

^b^QPS Netherlands BV, Groningen, The Netherlands

^c^Vast Therapeutics, Morrisville, North Carolina, USA

^d^University Hospital La Paz. IdiPAZ. Servicio Madrileño de Salud (SERMAS), Madrid, Spain

^e^University Medical Center, Dept of Clinical Pharmacy & Pharmacology, Groningen, The Netherlands

Running Title: TBAJ-587 First In Human (FIH) Trial in Healthy Adults

#Address correspondence to [jerry.nedelman@tballiance.org](mailto:jerry.nedelman@tballiance.org).

Contents

[**Figure S1. Study Participant Disposition**	3](#_Toc217034922)

[**Figure S2. Semi-logarithmic Scale of mean Pre-dose Plasma Concentrations of TBAJ-587 Following Multiple Doses of 50, 100, and 200 mg TBAJ-587 Oral Suspension in Healthy Adults under Fed Conditions from Day 2 through Day 28 for the MAD Cohorts.**	4](#_Toc217034923)

[**Figure S3. Semi-logarithmic scale of mean Pre-dose Plasma Concentrations of TBAJ-587-M3 Following Multiple Doses of 50, 100, and 200 mg TBAJ-587 Oral Suspension in Healthy Adults under Fed Conditions from Day 2 through Day 28 for the MAD Cohorts.**	5](#_Toc217034924)

[**Table S1. SAD and FE Pharmacokinetic Parameters for M2**	10](#_Toc217034925)

[**Table S2. SAD and FE Pharmacokinetic Parameters for M12**	10](#_Toc217034926)

[**Table S3. Dose Proportionality of PK Parameters of TBAJ-587 Following a Single Dose of 25, 50, 100, 200, 400, and 800 mg TBAJ-587 Oral Suspension in Healthy Adults under Fasted Conditions for the SAD Cohorts.**	11](#_Toc217034927)

[**Table S4. Dose Proportionality of PK Parameters of TBAJ-587-M3 Following a Single Dose of 25, 50, 100, 200, 400, and 800 mg TBAJ-587 Oral Suspension in Healthy Adults under Fasted Conditions for the SAD Cohorts.**	11](#_Toc217034928)

[**Table S5. Statistical Comparisons of Pharmacokinetic Parameters of TBAJ-587 Following a Single Oral Dose of 200 mg TBAJ-587 Oral Suspension in Healthy Adults under Fasted and Fed Conditions**	12](#_Toc217034929)

[**Table S6. Statistical Comparisons of PK Parameters of TBAJ-587-M3 Following a Single Oral Dose of 200 mg TBAJ-587 Oral Suspension in Healthy Adults under Fasted and Fed Conditions in the Food Effect Cohorts.**	12](#_Toc217034930)

[**Table S7. MAD Pharmacokinetic Parameters for M2**	13](#_Toc217034931)

[**Table S8. MAD Pharmacokinetic Parameters for M12**	14](#_Toc217034932)

[**Table S9. Dose Proportionality of PK Parameters of TBAJ-587 Following Multiple of 50, 100, and 200 mg TBAJ-587 Oral Suspension in Healthy Adults under Fed Conditions on Days 1 , 14, and 28 for the MAD Cohorts.**	15](#_Toc217034933)

[**Table S10. Dose Proportionality of PK Parameters of TBAJ-587-M3 Following Multiple Oral QD Doses of 50, 100, and 200 mg TBAJ-587 in Healthy Male and Female Subjects under Fed Conditions on Days 1, 14 and 28 - Part 2, MAD Cohorts.**	15](#_Toc217034934)

[**Table S11. Mean Metabolite-to-TBAJ-587 Ratios (% of Parent Compound) Following Multiple Oral QD Doses of TBAJ-587 in Healthy Male and Female Subjects under Fed Conditions - Part 2, MAD Cohorts**	16](#_Toc217034935)

[**Table S12. Breakfast Menus**	17](#_Toc217034936)

[**Table S13. Treatment Emergent Adverse Events Occurring in At Least Two Participants**	18](#_Toc217034937)

## **Figure S1. Study Participant Disposition**

## **Figure S2. Semi-logarithmic Scale of mean Pre-dose Plasma Concentrations of TBAJ-587 Following Multiple Doses of 50, 100, and 200 mg TBAJ-587 Oral Suspension in Healthy Adults under Fed Conditions from Day 2 through Day 28 for the MAD Cohorts.**

## **Figure S3. Semi-logarithmic scale of mean Pre-dose Plasma Concentrations of TBAJ-587-M3 Following Multiple Doses of 50, 100, and 200 mg TBAJ-587 Oral Suspension in Healthy Adults under Fed Conditions from Day 2 through Day 28 for the MAD Cohorts.**

**Figure S4. Dose Proportionality Plot of AUC_last_ vs Dose for TBAJ-587 Following a Single Oral Dose of TABJ-587 in Health Male and Female Subjects under Fasted Conditions – Part 1, SAD Cohorts**

**Figure S5. Dose Proportionality Plot of C_max_ vs Dose for TBAJ-587 Following a Single Oral Dose of TABJ-587 in Health Male and Female Subjects under Fasted Conditions – Part 1, SAD Cohorts**

**Figure S6. Dose Proportionality Plot of AUC_last_ vs Dose for TBAJ-587-M3 Following a Single Oral Dose of TABJ-587 in Health Male and Female Subjects under Fasted Conditions – Part 1, SAD Cohorts**

**Figure S7. Dose Proportionality Plot of C_max_ vs Dose for TBAJ-587-M3 Following a Single Oral Dose of TABJ-587 in Health Male and Female Subjects under Fasted Conditions – Part 1, SAD Cohorts**

## **Table S1. SAD and FE Pharmacokinetic Parameters for M2**

| **Arm** |  | **T_max_ (h)** | | **C_max_ (ng/mL)** | | **AUC_last_ (h*ng/mL)** | |
| --- | --- | --- | --- | --- | --- | --- | --- |
|  | **n** | **Median** | **Range** | **Mean** | **SD** | **Mean** | **SD** |
| **SAD (Oral Suspension, Fasting)** | | | | | | | |
| 25 mg | 6 | 5.00 | 4.02, 78.0 | 1.24 | 0.112 | 2.45 | 1.31 |
| 50 mg | 4 | 5.00 | 4.00, 6.00 | 2.68 | 0.360 | 58.1 | 71.9 |
| 100 mg | 5 | 5.00 | 5.00, 10.05 | 3.16 | 0.579 | 559 | 831 |
| 200 mg | 6 | 5.00 | 4.00, 6.00 | 4.80 | 1.54 | 342 | 441 |
| 400 mg | 6 | 5.00 | 5.00, 5.00 | 8.68 | 2.41 | 2300 | 1450 |
| 800 mg | 6 | 5.00 | 4.00, 10.00 | 14.7 | 4.17 | 5800 | 1880 |
| **Food Effect (Oral Suspension)** | | | | | | | |
| 200 mg fed | 9 | 8.00 | 5.00, 12.00 | 4.63 | 1.81 | 1450 | 1470 |
| 200 mg fasted | 8 | 5.01 | 4.00, 6.00 | 4.69 | 1.44 | 373 | 401 |

AUC_last_, area under the concentration-time curve from zero to last quantifiable sample collection time; Cmax, maximum (peak) plasma concentration; SD, standard deviation; T_max_, time to maximum (peak) plasma concentration.

## **Table S2. SAD and FE Pharmacokinetic Parameters for M3**

| **Arm** |  | **T_max_ (h)** | | **C_max_ (ng/mL)** | | **AUC_last_ (h*ng/mL)** | |
| --- | --- | --- | --- | --- | --- | --- | --- |
|  | **n** | **Median** | **Range** | **Mean** | **SD** | **Mean** | **SD** |
| **SAD (Oral Suspension, Fasting)** | | | | | | | |
| 25 mg | 6 | 14.00 | (5.00, 264.65) | 1.31 | 0.101 | 48.1 | 23.3 |
| 50 mg | 4 | 5.50 | (5.00, 16.00) | 2.66 | 0.704 | 590 | 716 |
| 100 mg | 5 | 12.00 | (10.00, 54.00) | 3.60 | 1.10 | 1080 | 1020 |
| 200 mg | 6 | 9.00 | (6.00, 12.00) | 5.30 | 2.40 | 3170 | 2230 |
| 400 mg | 6 | 10.00 | (8.00, 32.00) | 11.0 | 3.59 | 6390 | 2580 |
| 800 mg | 6 | 11.00 | (8.00, 12.00) | 19.2 | 5.46 | 11300 | 1980 |
| **Food Effect (Oral Suspension)** | | | | | | | |
| 200 mg fed | 9 | 28.00 | (10.00, 313.77) | 6.73 | 2.52 | 6490 | 2280 |
| 200 mg fasted | 8 | 9.00 | (6.00, 12.00) | 5.07 | 2.11 | 2870 | 2060 |

AUC_last_, area under the concentration-time curve from zero to last quantifiable sample collection time; Cmax, maximum (peak) plasma concentration; SD, standard deviation; T_max_, time to maximum (peak) plasma concentration.

## **Table S3. SAD and FE Pharmacokinetic Parameters for M12**

| **Arm** |  | **T_max_ (h)** | | **C_max_ (ng/mL)** | | **AUC_last_ (h*ng/mL)** | |
| --- | --- | --- | --- | --- | --- | --- | --- |
|  | **n** | **Median** | **Range** | **Mean** | **SD** | **Mean** | **SD** |
| **SAD (Oral Suspension, Fasting)** | | | | | | | |
| 25 mg | 0 | N/A | N/A | N/A | N/A | N/A | N/A |
| 50 mg | 0 | N/A | N/A | N/A | N/A | N/A | N/A |
| 100 mg | 0 | N/A | N/A | N/A | N/A | N/A | N/A |
| 200 mg | 0 | N/A | N/A | N/A | N/A | N/A | N/A |
| 400 mg | 3 | 78.0 | 5.0, 239.40 | 1.28 | 0.402 | 149 | 226 |
| 800 mg | 5 | 84.03 | 7.00, 263.53 | 1.55 | 0.328 | 292 | 212 |
| **Food Effect (Oral Suspension)** | | | | | | | |
| 200 mg fed | 2 | 185.92 | 155.30, 216.53 | 1.11 | 0.0141 | 105 | 40.7 |
| 200 mg fasted | 0 | N/A | N/A | N/A | N/A | N/A | N/A |

AUC_last_, area under the concentration-time curve from zero to last quantifiable sample collection time; Cmax, maximum (peak) plasma concentration; SD, standard deviation; T_max_, time to maximum (peak) plasma concentration.

## **Table S4. MAD Pharmacokinetic Parameters for M3**

| **Arm** |  | **T_max_ (h)** | | **C_max_ (ng/mL)** | | **AUC_tau_ (h*ng/mL)** | | **R_Cmax_** | | **R_AUC_** | | **T_1/2_ (h)** | |
| --- | --- | --- | --- | --- | --- | --- | --- | --- | --- | --- | --- | --- | --- |
|  | **n** | **Median** | **Range** | **Mean** | **SD** | **Mean** | **SD** | **Mean** | **SD** | **Mean** | **SD** | **Mean** | **SD** |
| **Day 1** | | | | | | | | | | | | | |
| **50 mg** | 6 | 10.0 | 7.00, 12.05 | 1.72 | 0.283 | 24.2 | NA | NA | NA | NA | NA | NA | NA |
| **100 mg** | 9 | 12.00 | 7.00, 16.00 | 3.83 | 1.63 | 56.6 | 18.1 | NA | NA | NA | NA | NA | NA |
| **200 mg** | 8 | 13.94 | 7.00, 23.92 | 6.37 | 2.76 | 128 | 77.3 | NA | NA | NA | NA | NA | NA |
| **Day 14** | | | | | | | | | | | | | |
| **50 mg** | 9 | 7.00 | 5.00, 12.00 | 14.3 | 3.85 | 307 | 93.8 | 8.34 | 1.52 | 11.6 | NA | NA | NA |
| **100 mg** | 9 | 7.00 | 5.00, 23.95 | 31.1 | 5.84 | 636 | 150 | 8.93 | 2.77 | 8.49 | 0.229 | NA | NA |
| **200 mg** | 9 | 5.00 | 5.00, 16.00 | 68.1 | 17.2 | 1510 | 396 | 11.9 | 4.25 | 9.43 | 0.812 | NA | NA |
| **Day 28** | | | | | | | | | | | | | |
| **50 mg** | 9 | 8.00 | 5.00, 12.02 | 23.7 | 6.37 | 491 | 139 | 14.3 | 2.27 | 19.7 | NA | 1800 | 993 |
| **100 mg** | 9 | 7.00 | 5.00, 16.00 | 53.8 | 10.1 | 1090 | 215 | 15.7 | 5.66 | 19.4 | 6.36 | 2460 | 1160 |
| **200 mg** | 9 | 8.00 | 0.52, 16.00 | 119 | 31.6 | 2440 | 762 | 21.3 | 7.38 | 18.7 | 5.77 | 1650 | 398 |

Sample sizes vary because some parameters could not be computed due to missing observations near Tmax, observations below quantification limit, profiles not meeting pre-specified quality criteria, or, for accumulation ratios, missing values on Day 1.

T_1/2_ reported only for Day 28 where sampling continued beyond 24 hours post dose.

AUCtau, area under the curve limited to the end of a dosing interval; CLss/F, apparent clearance, Cmax, maximum (peak) plasma concentration; NA, not applicable; RAUC, AUC accumulation ratio as compared to 1st fed day; RCmax, Cmax accumulation ratio as compared to 1st fed day; SD, standard deviation; T1/2, terminal-phase elimination half-life; Tmax, time to maximum (peak) plasma concentration.

## **Table S5. Dose Proportionality of PK Parameters of TBAJ-587 Following a Single Dose of 25, 50, 100, 200, 400, and 800 mg TBAJ-587 Oral Suspension in Healthy Adults under Fasted Conditions for the SAD Cohorts.**

| **Parameter^a^** | **Point Estimate (β)** | **90% Confidence Interval of β** |
| --- | --- | --- |
| C_max_ (ng/mL) | 0.788 | (0.676, 0.899) |
| AUC_last_ (h*ng/mL) | 0.941 | (0.813, 1.07) |
| Power Model: Y = α (Dose)^β^ Dose proportionality is considered to hold if the 90% confidence intervals of β for those parameters include the value of 1. ^a^: Dose proportionality was not performed on AUC_inf_ as only 1 subject in Cohort 3, 2 subjects in Cohort 5, and 2 subjects in Cohort 6 had evaluable AUC_inf_. | | |

## **Table S6. Dose Proportionality of PK Parameters of TBAJ-587-M3 Following a Single Dose of 25, 50, 100, 200, 400, and 800 mg TBAJ-587 Oral Suspension in Healthy Adults under Fasted Conditions for the SAD Cohorts.**

| **Parameter^a^** | **Point Estimate (β)** | **90% Confidence Interval of β** |
| --- | --- | --- |
| C_max_ (ng/mL) | 0.741 | (0.649, 0.833) |
| AUC_last_ (h*ng/mL) | 1.52 | (1.30, 1.74) |
| Power Model: Y = α (Dose)^β^ Dose proportionality is considered to hold if the 90% confidence intervals of β for those parameters include the value of 1. ^a^: Dose proportionality was not performed on AUC_inf_ as only 1 subject in Cohort 6 had evaluable AUC_inf_. | | |

## **Table S7. Statistical Comparisons of Pharmacokinetic Parameters of TBAJ-587 Following a Single Oral Dose of 200 mg TBAJ-587 Oral Suspension in Healthy Adults under Fasted and Fed Conditions**

|  | **Fed** | | **Fasted** | | **Fed/Fasted** | |
| --- | --- | --- | --- | --- | --- | --- |
| **Parameter^a,b^** | **n** | **GLSM** | **N** | **GLSM** | **GMR** | **90% Confidence Interval** |
| C_max_ (ng/mL) | 9 | 620 | 8 | 179 | 346 | (238, 503) |
| AUC_last_ (h*ng/mL) | 9 | 36500 | 8 | 16100 | 226 | (160, 321) |
| AUC_last_, area under the concentration-time curve from zero to last sample collection time; Cmax, maximum (peak) plasma concentration; GLSM, Geometric least squares mean; GMR, Geometric least squares mean ratio (reported as percentage).  ^a^: Back-transformed least squares mean and confidence interval from ANOVA model performed on log-transformed values.  ^b^: Statistical comparison was not performed on AUC_inf_ as only 2 subjects under fed conditions had evaluable AUC_inf_. | | | | | | |

## **Table S8. Statistical Comparisons of PK Parameters of TBAJ-587-M3 Following a Single Oral Dose of 200 mg TBAJ-587 Oral Suspension in Healthy Adults under Fasted and Fed Conditions in the Food Effect Cohorts.**

|  | **Fed** | | **Fasted** | | **Fed/Fasted** | |
| --- | --- | --- | --- | --- | --- | --- |
| **Parameter^a,b^** | **N** | **GLSM** | **N** | **GLSM** | **GMR** | **90% Confidence Interval** |
| C_max_ (ng/mL) | 9 | 6.34 | 8 | 4.68 | 135 | (96.4, 190) |
| AUC_last_ (h*ng/mL) | 9 | 6120 | 8 | 2100 | 291 | (161, 525) |
| GLSM=Geometric least squares mean; GMR=Geometric least squares mean ratio (reported as percentage)  ^a^: Back-transformed least squares mean and confidence interval from ANOVA model performed on log-transformed values.  ^b^: Statistical comparison was not performed on AUC_inf_ as only 1 subject under fed conditions had evaluable AUC_inf_. | | | | | | |

## **Table S9. MAD Pharmacokinetic Parameters for M2**

| **Arm** |  | **T_max_ (h)** | | **C_max_ (ng/mL)** | | **AUC_tau_ (h*ng/mL)** | | **R_Cmax_** | | **R_AUC_** | | **T_1/2_ (h)** | |
| --- | --- | --- | --- | --- | --- | --- | --- | --- | --- | --- | --- | --- | --- |
|  | **n** | **Median** | **Range** | **Mean** | **SD** | **Mean** | **SD** | **Mean** | **SD** | **Mean** | **SD** | **Mean** | **SD** |
| **Day 1** | | | | | | | | | | | | | |
| **50 mg** | 9 | 5.00 | 5.00, 12.05 | 1.43 | 0.318 | 9.64 | 4.70 | NA | NA | NA | NA | NA | NA |
| **100 mg** | 9 | 5.00 | 5.00, 8.02 | 3.16 | 1.33 | 36.6 | 18.1 | NA | NA | NA | NA | NA | NA |
| **200 mg** | 9 | 7.00 | 5.00, 8.02 | 6.32 | 2.80 | 27.9 | 9.29 | NA | NA | NA | NA | NA | NA |
| **Day 14** | | | | | | | | | | | | | |
| **50 mg** | 9 | 7.00 | 5.00, 7.92 | 5.58 | 1.29 | 106 | 26.7 | 4.03 | 1.16 | 12.2 | 4.22 | NA | NA |
| **100 mg** | 9 | 5.00 | 5.00, 8.00 | 12.0 | 3.33 | 221 | 54.8 | 4.23 | 1.64 | 6.36 | 1.99 | NA | NA |
| **200 mg** | 9 | 5.00 | 5.00, 16.00 | 29.4 | 7.90 | 617 | 157 | 5.06 | 1.63 | 6.68 | 1.69 | NA | NA |
| **Day 28** | | | | | | | | | | | | | |
| **50 mg** | 9 | 7.00 | 5.00, 16.00 | 9.94 | 2.07 | 202 | 46.5 | 7.19 | 1.89 | 22.8 | 7.94 | 2090^a^ | 1350 |
| **100 mg** | 9 | 5.00 | 5.00, 16.00 | 20.2 | 5.72 | 411 | 114 | 7.26 | 3.17 | 13.1 | 6.01 | 3040^b^ | 820 |
| **200 mg** | 9 | 5.00 | 2.00, 16.00 | 45.6 | 14.3 | 941 | 303 | 7.70 | 2.39 | 11.0 | 2.67 | 2700^c^ | 589 |

T_1/2_ reported only for Day 28 where sampling continued beyond 24 hours post dose. adjusted R^2^ value < 0.80: ^a^n=5; ^b^n=8; ^c^n=8.

AUC_tau_, area under the curve limited to the end of a dosing interval; CL_ss_/F, apparent clearance, C_max_, maximum (peak) plasma concentration; NA, not applicable; R_AUC_, AUC accumulation ratio as compared to 1^st^ fed day; R_Cmax_, Cmax accumulation ratio as compared to 1^st^ fed day; SD, standard deviation; T_1/2_, terminal-phase elimination half-life; T_max_, time to maximum (peak) plasma concentration.

## **Table S10. MAD Pharmacokinetic Parameters for M12**

| **Arm** |  | **T_max_ (h)** | | **C_max_ (ng/mL)** | | **AUC_tau_ (h*ng/mL)** | | **R_Cmax_** | | **R_AUC_** | | **T_1/2_ (h)** | |
| --- | --- | --- | --- | --- | --- | --- | --- | --- | --- | --- | --- | --- | --- |
|  | **n** | **Median** | **Range** | **Mean** | **SD** | **Mean** | **SD** | **Mean** | **SD** | **Mean** | **SD** | **Mean** | **SD** |
| **Day 1** | | | | | | | | | | | | | |
| **50 mg** | 0 | NA | NA | NA | NA | NA | NA | NA | NA | NA | NA | NA | NA |
| **100 mg** | 0 | NA | NA | NA | NA | NA | NA | NA | NA | NA | NA | NA | NA |
| **200 mg** | 1 | 24.0 | 24.0, 24.0 | 1.04 | NA | 4.22 | NA | NA | NA | NA | NA | NA | NA |
| **Day 14** | | | | | | | | | | | | | |
| **50 mg** | 6 | 8.00 | 6.00, 16.03 | 1.86 | 1.07 | 26.6^d^ | 8.87 | NA | NA | NA | NA | NA | NA |
| **100 mg** | 9 | 15.98 | 5.02, 23.92 | 3.31 | 1.28 | 108^e^ | NA | NA | NA | NA | NA | NA | NA |
| **200 mg** | 9 | 7.00 | 5.00, 23.93 | 8.34 | 2.80 | 185^f^ | 39.0 | NA | NA | NA | NA | NA | NA |
| **Day 28** | | | | | | | | | | | | | |
| **50 mg** | 9 | 7.00 | 3.00, 12.00 | 2.47 | 0.850 | 50.9 | 29.6 | NA | NA | NA | NA | 2610^a^ | 1830 |
| **100 mg** | 9 | 5.00 | 5.00, 16.00 | 6.81 | 3.02 | 142 | 64.9 | NA | NA | NA | NA | 1750^b^ | 582 |
| **200 mg** | 9 | 8.00 | 3.00, 16.00 | 15.9 | 6.68 | 330 | 151 | 27.3^e^ | NA | 146^e^ | NA | 1560^c^ | 252 |

T_1/2_ reported only for Day 28 where sampling continued beyond 24 hours post dose. adjusted R^2^ value < 0.80: ^a^n=7; ^b^n=7; ^c^n=7.

^d^ AUC_tau_ from 4 subjects could not be estimated because the sampling ended earlier than the end of doing interval and meanwhile the concentration curve could not be extrapolated due to less than three measurable concentrations after Cmax or positive slope in the terminal phase.

^e^ AUC_tau_ from 8 subjects could not be estimated because the sampling ended earlier than the end of doing interval and meanwhile the concentration curve could not be extrapolated due to less than three measurable concentrations after Cmax or positive slope in the terminal phase.

^f^ AUC_tau_ from 6 subjects could not be estimated because the sampling ended earlier than the end of doing interval and meanwhile the concentration curve could not be extrapolated due to less than three measurable concentrations after Cmax or positive slope in the terminal phase.

AUC_tau_, area under the curve limited to the end of a dosing interval; CL_ss_/F, apparent clearance, C_max_, maximum (peak) plasma concentration; NA, not applicable; R_AUC_, AUC accumulation ratio as compared to 1^st^ fed day; R_Cmax_, Cmax accumulation ratio as compared to 1^st^ fed day; SD, standard deviation; T_1/2_, terminal-phase elimination half-life; T_max_, time to maximum (peak) plasma concentration.

## **Table S11. Dose Proportionality of PK Parameters of TBAJ-587 Following Multiple of 50, 100, and 200 mg TBAJ-587 Oral Suspension in Healthy Adults under Fed Conditions on Days 1 , 14, and 28 for the MAD Cohorts.**

| **Parameter** | **Point Estimate (β)** | **90% Confidence Interval of β** |
| --- | --- | --- |
| *MAD Day 1* | | |
| C_max_ (ng/mL) | 0.833 | (0.618, 1.05) |
| AUC_tau_ (h*ng/mL) | 0.946 | (0.784, 1.11) |
| *MAD Day 14* | | |
| C_max_ (ng/mL) | 0.864 | (0.643, 1.09) |
| AUC_tau_ (h*ng/mL) | 0.920 | (0.725, 1.11) |
| *MAD Day 28* | | |
| C_max_ (ng/mL) | 0.833 | (0.640, 1.03) |
| AUC_tau_ (h*ng/mL) | 0.901 | (0.693, 1.11) |
| Power Model: Y = α (Dose)^β^  Dose proportionality is considered to hold if the 90% confidence intervals of β for those parameters include the value of 1. | | |

## **Table S12. Dose Proportionality of PK Parameters of TBAJ-587-M3 Following Multiple Oral QD Doses of 50, 100, and 200 mg TBAJ-587 in Healthy Male and Female Subjects under Fed Conditions on Days 1, 14 and 28 - Part 2, MAD Cohorts.**

| **Parameter** | **Point Estimate (β)** | **90% Confidence Interval of β** |
| --- | --- | --- |
| *MAD Day 1* | | |
| C_max_ (ng/mL) | 0.884 | (0.649, 1.12) |
| AUC_tau_ (h*ng/mL) | 1.09 | (0.429, 1.74) |
| *MAD Day 14* | | |
| C_max_ (ng/mL) | 1.13 | (0.997, 1.26) |
| AUC_tau_ (h*ng/mL) | 1.15 | (0.976, 1.33) |
| *MAD Day 28* | | |
| C_max_ (ng/mL) | 1.16 | (1.02, 1.30) |
| AUC_tau_ (h*ng/mL) | 1.15 | (0.995, 1.30) |
| Power Model: Y = α (Dose)^β^  Dose proportionality is considered to hold if the 90% confidence intervals of β for those parameters include the value of 1. | | |

## **Table S13. Mean Metabolite-to-TBAJ-587 Ratios (% of Parent Compound) Following Multiple Oral QD Doses of TBAJ-587 in Healthy Male and Female Subjects under Fed Conditions - Part 2, MAD Cohorts**

|  | | | **50 mg** | | **100 mg** | | **200 mg** | |
| --- | --- | --- | --- | --- | --- | --- | --- | --- |
| **Analyte** | **Parameter** | **Day** | **N** | **AM** | **N** | **AM** | **N** | **AM** |
| TBAJ-587-M2 | MR_Cmax_ | 1 | 9 | 0.975 | 9 | 0.976 | 9 | 1.29 |
|  |  | 14 | 9 | 2.13 | 9 | 2.52 | 9 | 3.31 |
|  |  | 28 | 9 | 3.14 | 9 | 3.63 | 9 | 4.56 |
|  | MR_AUCtau_ | 1 | 5 | 0.587 | 9 | 1.34 | 9 | 1.89 |
|  |  | 14 | 8 | 3.38 | 6 | 3.96 | 7 | 5.39 |
|  |  | 28 | 9 | 4.60 | 9 | 5.83 | 9 | 6.22 |
| TBAJ-587-M3 | MR_Cmax_ | 1 | 6 | 1.31 | 9 | 1.20 | 8 | 1.27 |
|  |  | 14 | 9 | 5.52 | 9 | 6.65 | 9 | 7.84 |
|  |  | 28 | 9 | 7.54 | 9 | 9.91 | 9 | 12.1 |
|  | MR_AUCtau_ | 1 | 1 | 1.92 | 4 | 1.98 | 3 | 2.38 |
|  |  | 14 | 7 | 10.3 | 6 | 10.8 | 6 | 13.2 |
|  |  | 28 | 9 | 11.3 | 9 | 15.8 | 9 | 16.4 |
| TBAJ-587-M12 | MR_Cmax_ | 1 | 0 | NA | 0 | NA | 1 | 0.116 |
|  |  | 14 | 6 | 0.707 | 9 | 0.749 | 9 | 0.973 |
|  |  | 28 | 9 | 0.811 | 9 | 1.27 | 9 | 1.72 |
|  | MR_AUCtau_ | 1 | 0 | NA | 0 | NA | 1 | 0.0772 |
|  |  | 14 | 2 | 0.727 | 1 | 1.20 | 3 | 1.67 |
|  |  | 28 | 9 | 1.22 | 9 | 2.13 | 9 | 2.38 |
| Note: Several MR_Cmax_ and MR_AUCtau_ values were not calculated because C_max_ and AUC_tau_ for certain metabolites could not be estimated. | | | | | | | | |

## **Table S14. Breakfast Menus**

Part 1 Food Effect

2 eggs fried in butter

2 strips of bacon or 30-gram Brie cheese

2 slices of toast with butter

4 ounces of hash brown potatoes

8 ounces of whole milk

Part 2 MAD

2 slices wholewheat bread

2-4 portions (20-40 gram) of low-fat margarine

1-2 portions (25-50 gram) of sweet spreads (e.g. jams, fruit sprinkles, apple syrup)

0-1 slices (0-20 gram) of cheese (48+)

0-1 portions (0-15 gram) of cheese spread

0-1 slices (0-120 gram) of meat (chicken, ham, smoked meat)

1 cup (200 ml) of semi-skimmed milk

1-2 cups (150 – 300 ml) of decaffeinated coffee or theine free tea with/without milk and sugar

**Table S15. Statistical Comparisons of PK Parameters of TBAJ-587 and M3 Under Different Meal Conditions**

a. TBAJ-587 on Day 1 Following a 200 mg TBAJ-587 Oral Dose in Healthy Male and Female Subjects - Part 2, MAD Cohort 3, Standard Breakfast vs. Part 1, Food Effect Cohort, High-Fat Breakfast

|  | **Standard Breakfast** | | **High-Fat Breakfast** | | **Standard Breakfast/High-Fat Breakfast** | |
| --- | --- | --- | --- | --- | --- | --- |
| **Parameter^a^** | **N** | **GLSM** | **N** | **GLSM** | **GMR** | **90% Confidence Interval** |
| Cmax (ng/mL) | 9 | 491 | 9 | 620 | 79.15 | (63.50, 98.65) |
| AUC0-24 (h*ng/mL) | 9 | 4660 | 9 | 5450 | 85.51 | (76.03, 96.18) |

b. TBAJ-587-M3 on Day 1 Following a 200 mg TBAJ-587 Oral Dose in Healthy Male and Female Subjects - Part 2, MAD Cohort 3, Standard Breakfast vs. Part 1, Food Effect Cohort, High-Fat Breakfast

|  | **Standard Breakfast** | | **High-Fat Breakfast** | | **Standard Breakfast/High-Fat Breakfast** | |
| --- | --- | --- | --- | --- | --- | --- |
| **Parameter^a^** | **N** | **GLSM** | **N** | **GLSM** | **GMR** | **90% Confidence Interval** |
| Cmax (ng/mL) | _8_b | 5.89 | 9 | 6.34 | 92.85 | (66.45, 129.74) |
| AUC0-24 (h*ng/mL) | _3_b,c | 112 | 9 | 92.1 | 121.57 | (73.89, 200.03) |

c. TBAJ-587 on Day 1 Following a 200 mg TBAJ-587 Oral Dose in Healthy Male and Female Subjects - Part 2, MAD Cohort 3, Standard Breakfast vs. Part 1, Food Effect Cohort, Fasted Condition

|  | **Standard Breakfast** | | **Fasted** | | **Standard Breakfast/Fasted** | |
| --- | --- | --- | --- | --- | --- | --- |
| **Parameter^a^** | **N** | **GLSM** | **N** | **GLSM** | **GMR** | **90% Confidence Interval** |
| Cmax (ng/mL) | 9 | 491 | 8 | 179 | 273.99 | (184.59, 406.71) |
| AUC0-24 (h*ng/mL) | 9 | 4660 | 8 | 2280 | 204.09 | (148.82, 279.90) |

d. TBAJ-587-M3 on Day 1 Following a 200 mg TBAJ-587 Oral Dose in Healthy Male and Female Subjects - Part 2, MAD Cohort 3, Standard Breakfast vs. Part 1, Food Effect Cohort, Fasted Condition

|  | **Standard Breakfast** | | **Fasted** | | **Standard Breakfast/Fasted** | |
| --- | --- | --- | --- | --- | --- | --- |
| **Parameter^a^** | **N** | **GLSM** | **N** | **GLSM** | **GMR** | **90% Confidence Interval** |
| Cmax (ng/mL) | _8_b | 5.89 | 8 | 4.68 | 125.68 | (86.15, 183.35) |
| AUC0-24 (h*ng/mL) | _3_b,c | 112 | 8 | 73.0 | 153.43 | (86.58, 271.91) |

GLSM=Geometric least squares mean; GMR=Geometric least squares mean ratio (reported as percentage)

^a^: Back-transformed least squares mean and confidence interval from ANOVA model performed on log-transformed values.

^b^: Cmax and AUCtau could not be reliably determined due to missing sample around Tmax for 1 subject in Cohort 3.

^c^: AUCtau could not be estimated because the sampling ended earlier than the end of doing interval and meanwhile the concentration curve could not be extrapolated due to less than three measurable concentrations after Cmax or positive slope in the terminal phase for 5 subjects in Cohort 3.

## Table S16. Grade 2 to 4 treatment emergent adverse events, CL-001 all parts

| **Adverse event** | **TBAJ-587 (n = 71)** | **Placebo (n = 21)** |
| --- | --- | --- |
| Ear and labyrinth disorders | 1 | 0 |
| Tinnitus |  |  |
| Gastrointestinal disorders |  |  |
| Vomiting | 0 | 1 |
| General disorders and administration site conditions |  |  |
| Influenza like illness | 1 | 0 |
| Infections and infestations |  |  |
| COVID-19 | 2 | 0 |
| Investigations |  |  |
| Amylase increased | 1 | 0 |
| Aspartate aminotransferase increased | 1 | 0 |
| Blood creatinine phosphokinase increased | 7 | 0 |
| Blood glucose increased | 0 | 1 |
| Lipase increased | 2 | 1 |
| Neutrophil count decreased | 1 | 0 |
| Metabolism and nutrition disorders |  |  |
| Hypophosphatemia | 4 | 1 |
| Nervous system disorders |  |  |
| Headache | 1 | 1 |
| Respiratory, thoracic and mediastinal disorders |  |  |
| Nasal congestion | 0 | 1 |
| Oropharyngeal pain | 0 | 1 |

## **Table S17. Treatment Emergent Adverse Events Occurring in At Least Two Participants**

***Part 1 (SAD)***

|  | **TBAJ-587**  **(25 mg)**  **(N=6)** | | | | | **TBAJ-587**  **(50 mg)**  **(N=4)** | | | | | **TBAJ-587 (100 mg) (N=5)** | | | | | **TBAJ-587 (200 mg) (N=6)** | | | | | | **TBAJ-587 (400 mg) (N=6)** | | | | | **TBAJ-587 (800 mg) (N=6)** | | | | | **Placebo (N=12)** | | | | | |
| --- | --- | --- | --- | --- | --- | --- | --- | --- | --- | --- | --- | --- | --- | --- | --- | --- | --- | --- | --- | --- | --- | --- | --- | --- | --- | --- | --- | --- | --- | --- | --- | --- | --- | --- | --- | --- | --- |
|  | **Event** | | **Subject** | | | **Event** | | | **Subject** | | **Event** | | | **Subject** | | **Event** | | | **Subject** | | | **Event** | | **Subject** | | | **Event** | | **Subject** | | | **Event** | | | **Subject** | | |
| **Preferred Term** | **E** | | **n** | | **%** | **E** | | | **n** | **%** | **E** | | | **n** | **%** | **E** | | | **n** | **%** | | **E** | | **n** | **%** | | **E** | | **n** | | **%** | **E** | | | **n** | | **%** |
| Back pain | 0 | 0 | | 0 | | | 0 | 0 | | 0 | | 0 | 0 | | 0 | | 0 | 0 | | | 0 | 0 | 0 | | | 0 | 3 | 3 | | 50 | | | 0 | 0 | | 0 | |
| Catheter site related reaction | 1 | 1 | | 16.67 | | | 1 | 1 | | 25 | | 1 | 1 | | 20 | | 0 | 0 | | | 0 | 0 | 0 | | | 0 | 0 | 0 | | 0 | | | 3 | 3 | | 25 | |
| Flatulence | 0 | 0 | | 0 | | | 0 | 0 | | 0 | | 0 | 0 | | 0 | | 0 | 0 | | | 0 | 0 | 0 | | | 0 | 0 | 0 | | 0 | | | 2 | 2 | | 16.67 | |
| Headache | 1 | 1 | | 16.67 | | | 2 | 2 | | 50 | | 1 | 1 | | 20 | | 0 | 0 | | | 0 | 0 | 0 | | | 0 | 1 | 1 | | 16.67 | | | 2 | 1 | | .33 | |

***Part 1 (FE)***

|  | **TBAJ-587** | | | **TBAJ-587** | | |
| --- | --- | --- | --- | --- | --- | --- |
|  | **(200 mg Fasted)** | | | **(200 mg Fed)** | | |
|  | **(N=8)** | | | **(N=9)** | | |
|  | **Event** | **Subject** | | **Event** | **Subject** | |
| **Preferred Term** | **E** | **n** | **%** | **E** | **n** | **%** |
| Nasopharyngitis | 0 | 0 | 0 | 2 | 2 | 22.22 |
| Oropharyngeal pain | 0 | 0 | 0 | 2 | 2 | 22.22 |

Note: [In the group '200 mg Fasted', the data of six subjects were obtained from the 200 mg group of the SAD part.]

***Part 2 (MAD)***

|  | **TBAJ-587** | | | | | | **TBAJ-587** | | | | | **TBAJ-587** | | | | |  | | | | |  |
| --- | --- | --- | --- | --- | --- | --- | --- | --- | --- | --- | --- | --- | --- | --- | --- | --- | --- | --- | --- | --- | --- | --- |
|  | **(50 mg)** | | | | | | **(100 mg)** | | | | | **(200 mg)** | | | | | **Placebo** | | | | |  |
|  | **(N=9)** | | | | | | **(N=9)** | | | | | **(N=9)** | | | | | **(N=9)** | | | | |  |
|  | **Event** | | | **Subject** | | | **Event** | | **Subject** | | | **Event** | | **Subject** | | | **Event** | | **Subject** | | |  |
| **Preferred Term** | **E** | | | **n** | | **%** | **E** | | **n** | | **%** | **E** | | **n** | | **%** | **E** | | **n** | | **%** |  |
| Arthralgia | | 1 | 1 | | 11.11 | | | 2 | 2 | 22.22 | | | 0 | 0 | 0 | | | 0 | 0 | 0 | | |
| Back pain | | 3 | 2 | | 22.22 | | | 0 | 0 | 0 | | | 0 | 0 | 0 | | | 1 | 1 | 11.11 | | |
| Blood creatine phosphokinase increased | | 0 | 0 | | 0 | | | 4 | 3 | 33.33 | | | 1 | 1 | 11.11 | | | 0 | 0 | 0 | | |
| COVID-19 | | 1 | 1 | | 11.11 | | | 2 | 2 | 22.22 | | | 1 | 1 | 11.11 | | | 0 | 0 | 0 | | |
| Catheter site related reaction | | 0 | 0 | | 0 | | | 2 | 2 | 22.22 | | | 1 | 1 | 11.11 | | | 0 | 0 | 0 | | |
| Cough | | 0 | 0 | | 0 | | | 1 | 1 | 11.11 | | | 0 | 0 | 0 | | | 2 | 2 | 22.22 | | |
| Diarrhoea | | 2 | 2 | | 22.22 | | | 1 | 1 | 11.11 | | | 1 | 1 | 11.11 | | | 0 | 0 | 0 | | |
| Fatigue | | 1 | 1 | | 11.11 | | | 1 | 1 | 11.11 | | | 1 | 1 | 11.11 | | | 2 | 2 | 22.22 | | |
| Headache | | 1 | 1 | | 11.11 | | | 7 | 5 | 55.56 | | | 2 | 2 | 22.22 | | | 3 | 3 | 33.33 | | |
| Muscle spasms | | 0 | 0 | | 0 | | | 1 | 1 | 11.11 | | | 3 | 3 | 33.33 | | | 0 | 0 | 0 | | |
| Nasopharyngitis | | 1 | 1 | | 11.11 | | | 0 | 0 | 0 | | | 3 | 3 | 33.33 | | | 1 | 1 | 11.11 | | |
| Oropharyngeal pain | | 0 | 0 | | 0 | | | 2 | 2 | 22.22 | | | 0 | 0 | 0 | | | 1 | 1 | 11.11 | | |

## Details on Grade 4 TEAEs

There were three Grade 4 TEAEs among participants on TBAJ-587 and none among participants on placebo. The three events were:

1) Part 1, SAD (fasted): hypophosphatemia (value 0.37 mmol/L, normal range 0.8-1.4 mmol/L) in a participant administered a single dose of 200 mg TBAJ-587. This event was first reported as a Grade 4 TEAE at 8 weeks into follow up (Day 56), improved to a Grade 2 TEAE (value 0.74 mmol/L) by Day 84, but increased again to a Grade 4 TEAE (value 0.44 mmol/L) by the end of the follow-up period (Day 126). The participant was asymptomatic, had no other concurrent TEAEs or clinically significant laboratory abnormalities, and the event was deemed unrelated to trial drug by the investigator.

2) Part 1, SAD (fasted): elevated blood CPK (value 12700 u/L, normal range <200 U/L) in a participant administered a single dose of 400 mg TBAJ-587. This event was first reported at 4 weeks after dosing (Day 28), improved to Grade 3 (1300 U/L) at 5 weeks after dosing and resolved at 8 weeks after dosing (Day 56). On Day 28, there was a simultaneous elevation of Grade 3 AST (value 302 U/L, normal range <40 U/L) and CPK-Mb (value 99 U/L, normal range <25 U/L) with the AST improving to Grade 1 and the CPK-Mb to normal at 5 weeks after dosing. No abnormalities in any other safety laboratory parameters were detected. The participant was asymptomatic, and the events were deemed unrelated to trial drug by the investigator.

3) Part 2, MAD (fed): elevated blood CPK (value 1590 U/L, normal range <200 U/L) in a participant treated with 100 mg TBAJ-587 QD for 28 days. This event occurred 2 weeks after the end of the dosing period (Day 42) and resolved after another 6 weeks (Day 84). On Day 42, there was a simultaneous elevation of CPK-Mb (value 40 U/L, normal range <25 U/L)]. The participant was asymptomatic with no other concurrent TEAEs or abnormalities in any other safety lab parameters, and the event of elevated CPK was regarded unrelated to trial drug by the investigator.

These three TEAEs met the Grade 4 criteria based on the value of the laboratory abnormalities; they did not appear to be either dose or time dependent.

## Details on Grade 3 TEAEs

There were 3 Grade 3 TEAEs among 3 participants in the placebo groups and 15 Grade 3 TEAEs among 13 participants in the TBAJ-587 groups. In the participants receiving placebo, all in the SAD part, the events were hyperglycemia (Day 7), increased lipase (Day 56), and hypophosphatemia (Day 84 ). In the SAD TBAJ-587 groups, there were 8 Grade 3 TEAEs among 7 participants:

- 2 events of increased CPK (25 mg on Day 84, 400 mg on Day 34);
- 2 events of hypophosphatemia (50 mg on Day 126, 100 mg on Day 13);
- 1 event of increased amylase (200 mg on Day 13);
- 1event of increased AST (400 mg on Day 28);
- 1 event of increased lipase (800 mg on Day 7);
- 1 event of neutropenia (400 mg on Day 126).

In the MAD TBAJ-587 groups, there were 7 Grade 3 TEAEs among 6 participants:

- 3 events of increased CPK (100 mg on Day 14 , a different participant on 100 mg on Day 42, 200 mg on Day 42 );
- 2 events of increased lipase (the same participant on 100 mg, first on Day 3, resolving on Day 14, then a second event on Day 126);
- 1 event of hypophosphatemia (50 mg on Day 168);
- 1 event of tinnitus (200 mg on Day 7).

## Details on CPK Elevations

CPK was of interest because of preclinical findings of muscle toxicity in rats. In the SAD, there were 5 participants with CPK elevations reported as TEAEs (1 each in the 25 mg, 50 mg, 200, and 400 mg cohorts, and 1 in the placebo group), mostly Grade 2 or 3, except for one Grade 4 elevation (12700 U/L, normal range <200 U/L) on Study Day 28 in one participant in the 400 mg cohort, which improved to 1300 U/L (Grade 3) on Day 34 and resolved by Study Day 56. In the MAD, there were 4 participants with CPK elevations reported as TEAEs (3 in the 100 mg cohort and 1 in the 200 mg cohort), all Grade 3 except for one Grade 4 elevation (4600 U/L) on Study Day 42 in one participant in the 100 mg cohort, which resolved by Study Day 84. All participants with CPK elevations were asymptomatic.

All but one of the TEAEs of CPK elevation occurred after dosing and were deemed unrelated to TBAJ-587. While the study protocol did not allow strenuous exercise during inpatient stay or the 3 days prior to a follow-up study visit, all these CPK elevations during follow-up were associated with some type of intense physical activity. The one exception was a Grade 3 event that occurred while on treatment on Study Day 14 in a participant receiving 100 mg in the MAD. The elevation resolved promptly without intervention. Two participants with CPK elevations showed mild elevations of CPK-MB, which also resolved promptly. These were the 2 participants with Grade 4 elevations in CPK (1 in the SAD and 1 in the MD), which were also associated with mild elevations in AST. Their cardiac troponin T was within the normal range at the time of the CPK and CPK-MB elevations and all other time points. These participants were without clinical symptoms, and there were no clinically significant ECG changes. It is therefore likely that the CPK-MB (and AST) elevations were of skeletal muscle origin and not indicative of myocardial injury.

## Details on Hypophosphatemia

Hypophosphatemia was of interest because it was also observed preclinically in rats. In the SAD, 4 participants were reported with TEAEs of hypophosphatemia (1 each in the 50 mg, 100 mg, and 200 mg cohorts, and 1 in the placebo group), mostly Grade 3, except for one participant with 2 events of Grade 4 decrease, 0.37 mmol/L and 0.44 mmol/L (normal range 0.8-1.4 mmol/L) on Study Days 56 and 126, respectively. In the MAD, one participant in the 50 mg cohort experienced a Grade 3 hypophosphatemia on Study Day 168. All participants with hypophosphatemia TEAEs were completely asymptomatic. More details are provided in the Supplementary Material.
